# The Treatment of Fractures

**Published:** 1907-02-09

**Authors:** W. Watson Cheyne

**Affiliations:** Senior Surgeon, King's College Hospital


### Hospital Clinics.

### THE TREATMENT OF FRACTURES.

By W. WATSON CHEYNE, C.B., F.R.S., F.R.C.S.(Eng.), Senior Surgeon, King's College Hospital.

(Abstract of Clinical Lectures specially reported for The Hospital.)

The treatment of fractures of long bones is important by reason of its difficulty, and of its seriousness to the patient. Disability may be due to mal-union, to non-union, or to stiffness of joints, tendons, and muscles in the neighbourhood of the broken bone. Deformity may be produced by the force that caused the fracture, and may be kept up and aggravated by muscular contraction and by the weight of the limb below the broken ends. The cases where muscular action is of primary importance are those where separation of the fragments tends to occur. The trouble in all these cases of deformity is primarily due to imperfect reduction of the fractured ends and insufficient retention of the fragments in position.

Inability to use the limb may be due to mal-union of the fragments, especially where the fracture is in the vicinity of a joint; but it is chiefly caused by adhesions of the tendons and muscles to their sheaths, or to each other, or to the bones at the seat of fracture. It may also be caused by adhesions in the neighbouring joints, atrophy of the muscles, etc. These troubles are all essentially due to the prolonged fixation of the injured parts, for it must be borne in mind that in a fracture the lesion is not confined to the bone itself, but also affects the soft tissues, the result being that blood and lymph are poured out into the soft tissues, and if they are not removed by movement and massage, they lead to development of fibrous tissue, which tends to bind the various parts together. Unless the broken ends are brought into accurate apposition at the time of the injury, or soon after, they will never come right subsequently, and a perfect result cannot be obtained; if, on the other hand, they are properly brought together at the time, it is in most cases comparatively easy to keep them in good position, and thus to prevent many of the bad after-results. In my opinion, the first and most important point to be considered in the treatment of recent fractures is whether, and in what way, the fractured ends of the bones can be brought into accurate apposition. It must be borne in mind that if such replacement is not carried out in the first instance no amount of extension of the limb afterwards, and no possible arrangement of splints and apparatus, will bring the bones into proper apposition.

By reduction of a fracture I mean an attempt to interlock the broken ends. The ends of the bones are never broken off smoothly; there are always projecting portions on both surfaces, and if only these can be fitted into each other the ends will frequently retain their position to a considerable and sufficient extent without much trouble or pressure. No doubt it is often a very difficult matter to obtain really accurate interlocking of the broken ends, owing to the obliquity of the surfaces and their tendency to slip apart, and also because the swelling of the soft parts around, and the depth at which the bone lies, prevent the surgeon from accurately feeling what he is about. Indeed, accurate apposition is in a good many cases impossible without an operation; and although such apposition is the thing to be aimed at, the question naturally arises in cases where it is evidently not possible to carry it out, whether it is in the particular instance essential. This depends very much on the nature of the fracture. Where a fracture extends into a joint, or is in the immediate vicinity of a joint, no pains should be spared to get accurate replacement of the fragments, otherwise there will subsequently be a great deal of restriction of movement in the joint, and often complete stiffness, owing to the altered lines of the joint surfaces. This applies more especially to fractures above the elbow joint, and also about the knee, ankle, and wrist. In fractures of the shaft of the long bones exact accuracy of apposition is not necessarily of such importance; fractures of the leg, more especially, often give a perfect functional result, even although the bones are not accurately in apposition. In fractures of the shaft of the femur, on the other hand, such apposition is important, because if the ends overlap, shortening, often to a marked degree, is very apt to occur.

*Muscular contraction* forms an obstacle to reduction. If overlapping of the fragments has once occurred, the contraction of the muscles, which is increased and kept up by the irritation of the broken ends, not only increases the deformity, but is very difficult to overcome when we attempt to reduce the fracture. In other cases, for example in fractures of the patella and olecranon, the muscular contraction causes separation of the fragments, and prevents them from being afterwards kept together.

*Interposition of material*, such as periosteum, between the broken ends renders the proper reduction of the fracture very difficult and sometimes impossible, but unless it is rectified non-union is very apt to occur.

*Rotation of the fragments* is also a great obstacle to accurate reduction. Dr. Griffiths, of Cambridge, has shown that most fractures are spiral, and not simply transverse or oblique, and the result is that as the force continues to act after the bone is broken, the spiral movement is continued, and not only does the lower fragment overlap the other, but it is rotated to a very serious extent. If, now, in reducing the fracture, attention be not paid to this point, the broken surfaces will not come properly into apposition and there will be no possibility of interlocking the ends; consequently, as soon as the extension is released, the muscles come into play and the overlapping recurs. This is a most important fact to bear in mind, especially in oblique fractures, for if some degree of interlocking is not obtained at the time, shortening of the limb will inevitably occur, as no amount of extension will bring or keep the lower fragment in position against the pull of the muscles.

*Comminution of the broken ends* is also another cause of difficulty in the proper setting of fractures; instead of having simply two surfaces which can be interlocked sufficiently to retain their position without much effort, one or other surface may be so irregular that no proper approximation is possible; and besides, some of the fragments may get in between the ends of the bones and prevent proper reposition.

*The small size of one of the fragments* is another obstacle to the proper reduction of a fracture. This, of course, is especially the case where the fracture is in the immediate neighbourhood of a joint, and where the one fragment is so small that one cannot grasp it properly and get the two ends in apposition, and yet in these particular cases it is most important to do so. Further, in such fractures, the difficulty is increased by the fact that usually there is a large amount of swelling present, which prevents one feeling and getting a hold of the small fragment.

The chief difficulties in retaining the fractured ends in position afterwards are:—

1. *Incomplete reduction* of the broken ends is the main cause of difficulty, but in some cases a better result may not be possible, at least by manipulation, while in others, even after proper reduction, the fractured ends are very apt subsequently to slip out of position. For example, in the case of oblique fractures, the broken surfaces are often comparatively smooth, and the interlocking of the ends, even when they are brought accurately together, is imperfect; so that often, in spite of careful reduction, displacement may occur during the application of the apparatus; and if this takes place it will frequently be found that in a short time, owing to the muscular action, marked overlapping of the fragments has occurred.

2. *The weight of the lower part* of the limb, unless carefully guarded against in putting up the fracture, will produce bending, and in the case of oblique fractures, unlocking of the fragments with subsequent overlapping of the ends. For instance, in cases of fracture of the tibia, the foot, unless properly supported, is apt to fall back and lead to a bending at the seat of fracture; or, in cases of fracture of the shaft of the femur, the weight of the distal part of the limb is very apt to produce rotation outwards.

3. *Where one fragment is short* and has a strong muscle attached to it, it is very apt to be tilted and rotated out of position unless attention is paid to bringing the longer fragments into line with it. The best examples of this trouble are fractures below the lesser trochanter of the femur, and fractures near the upper end of the radius.

### How to Reduce a Fracture.

In the first place, before commencing the necessary manipulation, it is essential to have all the retentive arrangements fully prepared, so that they may be applied at once when the parts have been brought into proper line. To pull a limb about, and then to allow the displacement to recur while the retentive apparatus is being got ready, is only to lacerate the parts twice without any corresponding advantage.

In the great majority of fractures, reduction should be effected under an anæsthetic. Complete relaxation of the muscles is a very important point, otherwise they form a formidable obstacle to reduction, and the necessary manipulations increase their contraction and thus render the accurate reduction of the fracture very difficult, or perhaps impossible. Assistance is also necessary wherever it can be obtained, the duty of the assistant being to make the necessary extension on the distal fragment, which must be done steadily and not in jerks; to see that the extension is being carried out in the proper direction; and, more especially, to see that the position of the limb as regards rotation is correct. The body must also be fixed, and, wherever it is possible, a second assistant (who need not, however, be a skilled one) should be obtained for this purpose; the important point being that the surgeon should have both hands free to manipulate the fragments into position. Proper extension and counter-extension having been obtained, the surgeon proceeds to *manipulate* the fragments and to try to interlock them as accurately as possible.

In the first place, he has to see that nothing intervenes between the broken ends. This he judges of by the completeness of the crepitus, taking care, however, in testing this that he does not grind the surfaces together, and so break off any irregularities which will be of use in the way of interlocking the ends. Having made sure of this point, he tries to get the ends into accurate position by rotation and lateral movements, while steady extension is being carried out by the assistant. Theoretically this sounds simple, practically it is not at all easy. To feel the bones and to make sure that they are really in position is especially difficult in a fleshy part like the thigh. Hence, wherever it is possible, it is well to perform the reduction under the $x$-ray screen, so that the surgeon can see as well as feel whether the bones are in proper apposition; and this is especially important in cases of fractures about joints, where accuracy is very necessary. It is not, of course, by any means possible to carry this out in all cases, partly on account of the absence of the necessary apparatus, and partly on account of the expense; and, unless the matter is a very important one, the surgeon cannot leave the patient in pain while the apparatus is being obtained and fitted up. In any case, however, it is important to have $x$-ray photographs taken as soon as possible after the fracture has been put up, and these skiagrams should always be stereoscopic. This should be done while the injured parts are still soft, so that if any alteration in position seems necessary, it can still be carried out. If it is left for a week or two the tissues by that time have become so rigid and fixed that no proper rectification is possible without an open operation.

*The question of operation for reduction.* Should it be found impossible for some reason or other to get the bones into accurate apposition, the question as to what should be done must be at once considered, that is to say, one must decide whether to be content with the best position obtainable by manipulation, or whether further steps should be taken to secure a more perfect result. This raises the question of operation on recent fractures, for under these circumstances the difficulty cannot as a rule be overcome in any other way. In considering this question, two points must be borne in mind; in the first place, operations of this kind are of a severe and serious nature. The severity may be considerable, especially in the case of deep-seated bones, for in such circumstances an extensive incision must as a rule be employed in order to gain good access to the parts, and this involves considerable loss of blood, as well as a more or less prolonged operation, before the ends of the bones are satisfactorily fixed. The seriousness arises from the risk of sepsis, for if suppuration occurs in the wound disaster is very likely to follow; the various general septic diseases may set in, there may be necrosis of the ends of the bones, prolonged suppuration, and so on. Hence, unless the operator is certain of his aseptic work, he should not operate in cases of recent fracture. In the second place, even supposing that the surgeon is sure as regards asepsis, it must be remembered that some of the methods of fixation employed in these cases are not very satisfactory; and it is especially essential that the fixation should be firm, because after these operations there is always a good deal of bloody oozing, sometimes necessitating several changes of dressings before the wound has healed. This implies a good deal of movement, with the risk of displacement unless the parts are firmly fixed, and the assistance therefore of one or more individuals at each dressing. Thus, in deciding the question of operation, one has to consider the severity of the operation, the risk of sepsis, the firmness of the fixation, and whether the necessary assistance in the subsequent dressings is likely to be forthcoming. On the other hand, absolute apposition of the fragments is by no means always necessary for a satisfactory functional result; and a little overlapping of the fragments, which may look very bad in a skiagram, may not affect the functional result at all.

Hence a good deal of judgment must be exercised in deciding the question of open operation in cases of recent fracture, and before undertaking it the surgeon must be satisfied that if it is not done the functional result will be bad; that by the operation he can distinctly improve matters, and that it can be performed with safety. In regard to this question of operation, if it is to be done it should be within three or four days of the accident, otherwise the tissues become so infiltrated and firm that it is extremely difficult to get the ends of the bones into position, and more extensive incisions than are altogether desirable may be necessary to effect this object. In certain cases it is most desirable, where possible, to operate. Such are injuries where the fragments become separated by muscular action, and where soft tissues get in between the ends of the bones; such as fractures of the patella and olecranon. In fractures involving joints, with displacement of fragments, operation is also very necessary. This is more especially the case in fractures about the elbow-joint, particularly when the lower end of the humerus is broken up into two or more pieces. Other examples are fractures through, or just above, the condyles of the femur, if it be found impossible otherwise to get or retain the fragments in position. T-shaped fractures into joints almost always require operation. Fractures of the tibia where the lower end of the upper fragment tends to tilt forward and perforate the skin, or conversely, where it is found impossible to keep the heel forwards satisfactorily, should be fixed by operation. Where pieces are broken off from bones, as in fracture of the great trochanter of the femur, or of the great tuberosity of the humerus, operation may also be necessary to fix them in position.

More difficult is the question of operation on deep-seated bones such as the femur, and on double bones, such as the bones of the forearm. Operations on the shaft of the femur are difficult and extensive on account of the depth of the bone, the large incision required, and the risk of sepsis if great care is not taken. On the other hand, such fractures are often oblique and the fragments difficult to interlock in the first instance and to retain in position afterwards; thus marked overlapping of the fragments with consequent shortening is very apt to occur even in spite of the use of as great a degree of extension as can be borne. There are, no doubt, many cases of fracture of the femur where the results are not satisfactory, especially from the point of view of shortening. Hence it seems to me that the proper plan in these and similar fractures is to watch the case very carefully, to take frequent measurements and skiagrams, and if in a few days it is evident that the result is not likely to be satisfactory, to cut down before the parts have become so matted together that they will not stretch. The question of the forearm is also a difficult one. The proper fixation of the two bones by operation is by no means an easy matter; while, on the other hand, the fracture can be seen very readily and the ends of the bone brought into position under the *x*-rays. Here, again, a good deal will depend on the progress of the case, and except in rare instances I should not advocate operation in the first instance.

Much depends on the manner of fixation employed. Except for the patella and olecranon, wires are of little use, and the means I employ are plates, screws, and pegs. In such cases as detachment of the tuberosity of the humerus, screws or pegs, preferably the former (ordinary unplated steel screws), are the best. About the elbow, small metal or ivory pegs or nails are most frequently useful. But wherever the parts are large enough to permit of it, I use an aluminium plate embracing the two fragments and fastened on with tacks or small screws. The aluminium plates which I employ are pieces of sheet aluminium which can be readily bent but are not too thin, and are perforated with a number of small holes. The fractured ends having been laid bare on one side for at least an inch above and below the line of fracture, a piece of aluminium is cut and folded about one-third of the way round the bone, so as to project about an inch (or longer, if possible) above and below the fracture. The plate need not surround the bone to a greater extent, as long as it is well curved and securely nailed on. In most bones, unless they are very dense, ordinary tin-tacks or thin nails can be driven in and will hold the plates; in dense bones, and also if the plate is employed for old, mal-united fractures, the tin-tacks will not penetrate the dense bone, and it will be necessary to bore holes and put in small screws. Once the plate is securely fixed one can handle the limb readily without any fear of displacement; the wound is stitched up without any drainage tube, and in the great majority of cases the plate gives no further trouble. I have used this method in a considerable number of cases where bones have either been divided intentionally or accidentally, and am extremely satisfied with the firm fixation thus obtained; but, of course, in certain parts—such as the condyles of the humerus —the plate is not suitable, and one must be content with pegs or nails. In cases of fracture of two bones I have now on two or three occasions—for example, in the case of the fibula—where there has been an oblique fracture and a long splinter running down, pushed the long splinter into the medulla of the other fragment, and in this way obtained absolute steadiness and perfect union without any sign of the line of fracture subsequently; and I think this method is well worth bearing in mind, and might be extended with advantage in a considerable number of cases.

Having effected the reduction of the fracture, either with or without operation, suitable retentive apparatus must be applied. This varies, of course, for each fracture, but the guiding principle is the following: The splints are meant to retain the fractured ends of the bones in their proper position after these have been reduced and correctly fixed. They are not supposed to rectify an imperfectly corrected displacement by constantly forcing or pulling the bones into position. Thus is a very important point. If the fractured ends have been more or less satisfactorily interlocked, very little pressure is required to keep them in position, and consequently a splint may be put on so as to be comfortable to the patient and without any special pressure being applied; that is to say, the splints are used to keep the bones in line and prevent the displacement from mechanical causes, rather than to keep them forcibly in apposition and violently oppose muscular contraction.

The question of massage in the after-treatment of fractures has been much discussed, and more or less general agreement has been come to. It is quite evident that the sooner movements and massage can be begun, the quicker will the function of the limb be restored, so long as no displacement of the bones occurs during the manipulations. My own belief is that the fractured ends ought to be left quite still for eight or ten days after the injury, except in the case of fractures of the forearm, where the fingers can be left out of the splints and moved from the first. By that time the bones will be more or less stuck together with fairly firm material, so that a certain amount of force would be necessary to displace them; and from that time onwards the splints may be taken off once or twice a day, and massage and careful active and passive movements of the neighbouring joints and muscles carried out so as to prevent the occurrence of stiffness. In most cases, provided the patient does not put any strain on the part, the splints may be left off entirely in from three to four weeks. I do not approve of treating fractures without any splints, or of looking on massage as of primary importance, and reduction and retention as of quite minor value.

